# Comparison of Adjunctive Naoxintong versus Clopidogrel in Volunteers with the CYP2C19*2 Gene Mutation Accompanied with Qi Deficiency and Blood Stasis Constitution

**DOI:** 10.1155/2011/207034

**Published:** 2011-03-01

**Authors:** Hui Chen, Guangwei Yu, Hong Sun, Xiaoying Wu, Huan Wang

**Affiliations:** ^1^Department of Internal Medicine, Fujian Provincial Cardiovascular Disease Institute, Provincial Clinical College of Fujian Medical University, Fuzhou, Fujian 350001, China; ^2^Clinical Discipline of Chinese and Western Integrative Medicine, Fujian University on Traditional Chinese Medicine, Fuzhou 350108, China; ^3^Department of Pharmacy, Fujian Provincial Hospital, Fuzhou 350001, China

## Abstract

This study was to determine the impact of adjunctive Buchang Naoxintong Jiaonang (BNJ) to clopidogrel on volunteers with the CYP2C19*2 gene mutation accompanied with qi deficiency and blood stasis (QDBS) constitution. Eighteen males with QDBS constitution were selected, who were 6 CYP2C19*1/*1, 6 CYP2C19*1/*2, and 6 CYP2C19*2/*2, and signed informed consent. Results showed that the maximal platelet aggregation (Agg_max_) and 5 min aggregation (Agg_late_) with 5-*μ*mol/L ADP in three different CYP2C19*2 genotypes were significantly decreased after any drug therapy compared with corresponding baseline measurements (all values *P* < .05). But percent inhibitions of Agg_max_ and Agg_late_ (IPAs) in CYP2C19*2/*2 genotype at 4 hours, 24 hours, 3 days, and 7 days after clopidogrel administration were all the lowest among three CYP2C19*2 genotypes (*P* < .01), and IPAs in CYP2C19*1/*2 genotype were between CYP2C19*1/*1 and CYP2C19*2/*2. IPAs had no significant difference among three different CYP2C19*2 genotypes after BNJ or adjunctive BNJ. In addition, changes of CD62P, PAC1, and sCD40L were similar to changes of ADP-induced platelet aggregation in three different CYP2C19*2 genotypes. Conclusion was that adjunctive BNJ to clopidogrel can enhance the antiplatelet effect in volunteers with the CYP2C19*2 gene mutation.

## 1. Introduction

The recent US Food and Drug Administration (FDA) “boxed warning” on clopidogrel is based on the concern that the antiplatelet effect of clopidogrel depends primarily on its activation by the cytochrome P450 (CYP) system [1]. Patients with decreased CYP2C19 function because of genetic polymorphisms metabolize clopidogrel poorly and have higher rates of cardiovascular events after acute coronary syndrome (ACS) and percutaneous coronary interventions (PCIs) than patients with normal CYP2C19 function [2, 3]. CYP2C19*2 is the most common genetic variant reproducibly associated with variability in clopidogrel active metabolite bioavailability, antiplatelet effects, and clinical outcomes [4, 5]. To overcome deficits in clopidogrel responsiveness, one approach is to increase the dose of clopidogrel [6, 7]. Other strategy is to add a third drug (such as Cilostazol) to aspirin and clopidogrel to further enhance platelet inhibition [8]. The other approach is to substitute a newer, more potent platelet inhibitor drug (such as prasugrel) for clopidogrel [9]. However, these strategies that enhanced response to clopidogrel are often associated with a higher risk for bleeding that may be attributed to the overmuch inhibition of the TXA2 and ADP platelet activation pathways that are essential for normal hemostasis [10, 11]. These considerations underscore the need for agents that provide more comprehensive platelet inhibition without interfering with hemostasis, for greater protection against thrombotic events with no incremental bleeding risk. In traditional Chinese medicine (TCM), it is expressed by regulating the yin and yang balance [12], by applying holistic approaches to enhance the system's harmony, a method called dual regulation.

Qi deficiency and blood stasis syndrome is the most common syndrome in patients with coronary heart disease, especially after PCI [13, 14]. Buyang Huanwu decoction (BYHWD), a TCM formula, has been recognized as a treatment for coronary heart diseases with Qi deficiency and blood stasis syndrome and cerebrovascular diseases in clinic [15]. Buchang Naoxintong Jiaonang (BNJ) consists of BYHWD plus Scorpio and Hirudo. Therefore, it was a compound preparation of BYHWD. BNJ is an approved TCM for stroke [16], which is widely used, and is well tolerated. BNJ combined with aspirin could enhance the antiplatelet effect in patients with cardio-cerebrovascular diseases [17].

The purpose of this study was to determine the impact of adjunctive BNJ in volunteers with the CYP2C19*2 gene mutation accompanied with qi deficiency and blood stasis constitution during treatment of clopidogrel.

## 2. Methods

### 2.1. Subject Population

Male volunteers were eligible for enrollment if they were between 25 and 55 years of age, undergoing medical examination in Fujian Provincial Hospital, and identified as having genotype CYP2C19*2. Based on the theory of constitution of TCM [18, 19], qi deficiency constitution and blood stasis (or stagnant blood) constitution were evaluated. Qi deficiency constitution means insufficiency of primordial QI; lassitude, short breath, and spontaneous perspiration are its main characteristics. Blood stasis constitution means an impeded blood flow; dark or purplish tongue and complexion are its main characteristics. Major exclusion criteria included active bleeding and bleeding diatheses, any antiplatelet or anticoagulation therapy, contraindication to antiplatelet therapy, leukocyte count <3.0 × 10^9^/L, platelet count 100.0 < × 10^9^/L, aspartate aminotransferase or alanine aminotransferase levels >3 times upper normal, serum creatinine level >2.5 mg/dL, having no histories such as cardiovascular or cerebral vessels diseases, diabetes, phlebothrombosis, arterial thrombus, gastritis, peptic ulcer, hepatitis, cholecystitis, renal disease, neoplastic disease, external injury or operation within 6 months, inability to follow the protocol. The Institutional Review Board of Provincial Clinical College of Fujian Medical University approved the study protocol, and the patients provided written informed consent for participation. The study was performed in the Clinical Pharmacological Base of Fujian Provincial Hospital. CYP2C19*2 genotyping, adenosine diphosphate- (ADP-) induced platelet aggregation, enzyme immunoassays, and flow cytometry were finished in Fujian Provincial Key Laboratory of cardiovascular disease and Fujian Provincial Clinical Laboratory, respectively.

### 2.2. Genotyping by TaqMan Polymerase Chain Reaction (PCR)

Genomic DNA was extracted from 200 *μ*L peripheral potassium ethylenediaminetetraacetic acid-anticoagulated blood with the TIANamp Blood DNA Kit {TIANGEN BIOTECH (BEIJING) CO., LTD} according to the manufacturer's instruction. CYP2C19*2 (681G > A; rs4244285) was genotyped using polymerase chain reaction and restriction fragment length polymorphism (PCR-RFLP). MultiGene Gradient Thermal Cycler (TC9600-G-230V, Labnet International, Inc.) was selected. PCR was performed in a 25 *μ*L reaction mixture containing 1.0 *μ*L of DNA, 0.8 *μ*L of each primer (forward primer 5′-CAGAGCTTGGCATATTGTATC-3′ and reverse primer 5′-GTAAACACACAACTAGTCAATG-3′), 2.0 *μ*L of each dNTPs, 2.5 *μ*L of 10 reaction buffer, 17.6 *μ*L of sterilizing, and 0.3 *μ*L of Taq DNA polymerase (Xiamen Tagege Biotechnology Co., Ltd.). PCR parameters consisted of an initial denaturation for 5 min at 94°C, followed by 35 cycles of 30 s at 94°C, 20 s at 56°C, and 20 s at 72°C, and by a final extension for 5 min at 72°C. The PCR product was then digested with *SmaI *(Xiamen Tagege Biotechnology Co., Ltd.) for 5-6 hours at 30°C yielding 2 fragments of 212 and 109 bp in the case of the GG genotype. For the AA genotype a single band of 321 bp was observed. For the GA genotype 3 fragments of 321, 212, and 109 bp were observed. Genotypes were directly observed with an ImageMaster VDS-CL (Amersham Pharmacia Co., Sweden) and were confirmed with ABI 3700 Automated DNA Sequencer. PCR-RFLP was performed to genotype CYP2C19*2 in 360 medical examination male volunteers aged 25–55. There were 204 subjects with CYP2C19*1/*1(681GG) genotype, 127 subjects with CYP2C19*1/*2(681GA) genotype and 24 subjects with CYP2C19*2/*2(681AA) genotype.

### 2.3. Study Design

**Figure 1:**
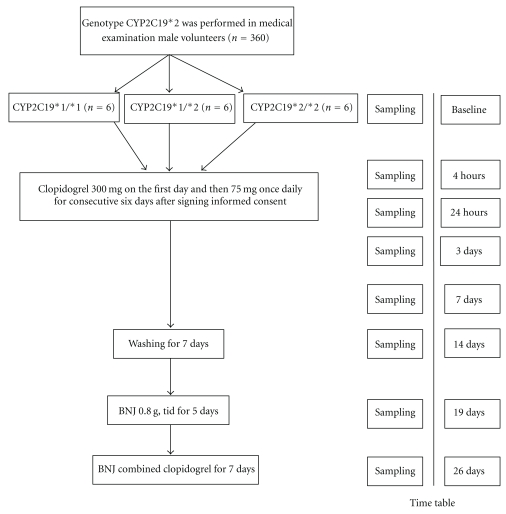
Study protocol timeline. The figure shows the ABNJC-CYP2C19*2 trial protocol.

The ABNJC-CYP2C19*2 (Adjunctive Buchang Naoxintong Jiaonang versus Clopidogrel in Volunteers with CYP2C19*2 Gene Mutation) study is a prospective, controlled platelet function study of CYP2C19*2 Gene Mutation in Volunteers with Qi Deficiency and Blood Stasis Constitution. The flow diagram of the study is depicted in Figure 1. Eighteen male volunteers with qi deficiency and blood stasis constitution were selected, who were 6 CYP2C19*1/*1, 6 CYP2C19*1/*2, and 6 CYP2C19*2/*2, and signed informed consent. All subjects have stopped taking any medicine two weeks before the study. There were no cigarette smoking, no drinking alcohol, and no drinking coffee during the study. Each subject took clopidogrel 300 mg on the first day and then 75 mg once daily for consecutive six days. After seven washing days, BNJ (0.8 g thrice per day) was taken for five days. Then BNJ-combined clopidogrel were taken for 7 days (the dose was the same as above). BNJ (Naoxintong) 0.4 g capsules (Compilation of The National Standard of Chinese Traditional Medicine no. WS-10001 (ZD-0001)-2002; Med-drug Permit no. Z20025001) were supplied by the Buchang Pharmaceutical Co., Ltd., and Plavix (clopidogrel bisulfate) 75 mg tablets (Med-drug Permit no. J20080090) were provided by Sanofi-Winthrop Industrie. The test, dispensing, and records were taken by GCP (good clinical practice) trained doctor. Venous blood samples were drawn by trained nurses. Blood samples were obtained to measure the ADP-induced platelet aggregation by turbidimetry at baseline and 4 hours, 24 hours, 3 days and 7 days after clopidogrel administration, 7 days after washing, 5 days after BNJ administration, and 7 days after BNJ combined clopidogrel, and measure the platelet count, platelet functional (ADP-induced platelet aggregation, Platelet activation of the membrane marker P-selectin (CD62p), and Platelet Activator combined-l (PAC-1) with flow cytometry) and inflammatory index (Soluble CD40L (sCD40L) with enzyme-linked Immunosorbent assay) at baseline, 7 days after clopidogrel administration, 5 days after BNJ administration, and 7 days after BNJ-combined clopidogrel, respectively. Hemoglobin (Hb), Platelet count, fasting plasma glucose (FSG), alanine aminotransferase (ALT), aspartate aminotransferase (AST), plasma total cholesterol (TC), and plasma creatinine (Cr) were remeasured at baseline and the end of the test, respectively. Creatinine clearance rate (CGCCr) was calculated by Cockcroft/Gault formula: CGCCr = (140-age)∗(Wt in kg)∗(0.85 if female)/(72∗Cr).

### 2.4. Adenosine Diphosphate-Induced Platelet Aggregation

Aggregation studies were performed within 2 hours after blood collection, at 37°C, by using a turbidimetric method of Born [20] on an LBY-NJ4-channel platelet aggregation analyzer (Beijing Precil Instrument Co., Ltd). The whole blood was centrifuged at 700 rpm for 4 min to prepare platelet-rich plasma (PRP). The remaining blood was further centrifuged at 3500 rpm for 10 min to prepare platelet-poor plasma (PPP). The platelet counts of PRP were adjusted to 200 × 10^9^/L. Results were recorded as light transmission at maximal aggregation (Agg_max_) and 5 min aggregation (Agg_late_) after the addition of ADP (SIGMA-ALORICH) at final concentrations of 5 *μ*mol/L. Agg_max_ is considered to reflect the activity of both P2Y1 and P2Y12 receptors, whereas Agg_late_ is more reflective of P2Y12 receptor activity. Inhibition of platelet aggregation (IPA) was defined as the percent decrease of aggregation values (Agg_max_ and Agg_late_) between baseline and after treatment and calculated as follows: IPA (%) = ([intensity of aggregation at baseline − intensity of aggregation after treatment]/[intensity of aggregation at baseline]) × 100 [8]. Percentage of platelet disaggregation between Agg_max_ and Agg_late_ was defined as follows: disaggregation (%) = ([Agg_max_ − Agg_late_]/[Agg_max_]) × 100 [8].

### 2.5. Flow Cytometry

All flow cytometric studies were conducted on Beckman Coulter Epics XL Flow Cytometer (Becton Dickinson, America) using CellQuest software (Becton Dickinson) for data acquisition and analysis. Analyses were performed on citrated whole blood diluted 1 : 9 in phosphate-buffered saline incubated with either PAC-1 fluorescein isothiocyanate-conjugated monoclonal antibody (Becton Dickinson) and anti-P-selectin (CD62p) phycoerythrin-conjugated monoclonal antibody (Becton Dickinson). Platelet activation markers (CD62p and PAC-1) were measured in unstimulated platelets and after stimulation of platelets with ADP (5 *μ*mol/L final concentration).

### 2.6. Enzyme Immunoassays (EIAs)

Serum levels of Soluble CD40 ligand (sCD40L, R&D Systems Inc., America) were determined by EIA (detection limit, 0.03 ng/mL; Bender Medsystems, Vienna, Austria) according to the manufacturer's instructions (intra- and interassay coefficient of variation <10%). Analysis was performed in duplicates in a blinded fashion. All samples from a given patient were analyzed in the same microtiter plate to minimize run-to-run variability.

### 2.7. Statistical Analysis

Data were analyzed using SPSS (version 13.0, SPSS Inc., America) and expressed as mean and SD. A paired samples *t*-test was used to make pairwise comparisons between baseline and each of treatment at each time point. The effects of adjunctive BNJ versus clopidogrel on platelet count, inhibition of platelet aggregation, platelet disaggregation, platelet activation, and inflammation marker in the three *CYP2C19 *genotype groups were compared by an analysis of variance. Multiple comparisons used LSD or Tamhane. A value of *P* < .05 was considered to indicate statistical significance.

## 3. Results

### 3.1. Subjects Characteristics

Baseline BP, BMI, Hb, platelet count, blood biochemical indicators, and CYP2C19*2 genotypes were performed in 360 medical examination male volunteers. Eighteen male volunteers with qi deficiency and blood stasis constitution were selected, who were 6 CYP2C19*1/*1, 6 CYP2C19*1/*2, and 6 CYP2C19*2/*2 (Table 1). At baseline there were no significant difference in platelet count, Agg_max_ and Agg_late_ with 5-*μ*mol/L ADP stimuli, CD62P, PAC-1, and sCD40L among three different CYP2C19*2 genotypes (Tables 2 and 3). 

All subjects completed the treatment allocated in the study protocol. All treatments were well tolerated, and no subject discontinued the study drugs. Compared with baseline, Hb, platelet count, blood biochemical indicators at the end of the test had no significant difference in three different CYP2C19*2 genotypes, except for CGCCr in CYP2C19*1/*1 genotype (Tables 3 and 4). 

### 3.2. ADP-Induced Platelet Aggregation

**Figure 2:**
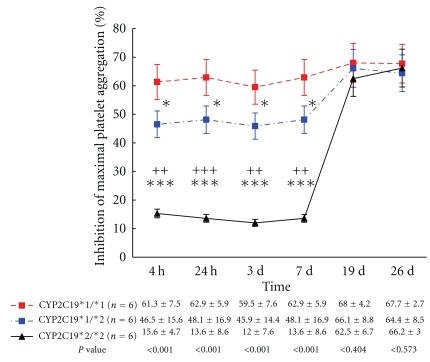
Inhibition of maximal platelet aggregation with 5-*μ*mol/L ADP between baseline and after therapy among the three *CYP2C19 *genotypes. The points represent the mean platelet inhibition. Bars indicate standard deviations. 4 h, 24 h, 3 d, and 7 d was after clopidogrel therapy. 19 d was 5 days after BNJ administration. 26 d was 7 days after adjunctive BNJ. The *P* values are the results of the one-way analysis of variance. “∗” symbol expresses that the *P* values are for comparison to CYP2C19*1/*1 in LSD or Tamhane. ∗, ∗∗, ∗∗∗,represent *P* < .05, <.01, and <.001, respectively; “+” symbol expresses that the *P* values are for comparison to CYP2C19*1/*2 in LSD or Tamhane; +, ++, +++ represent *P* < .05, <.01, and <.001, respectively.

The results of a paired samples *t*-test showed that subjects in three different CYP2C19*2 genotypes experienced a definite reduction in Agg_max_ after any drug (clopidogrel or BNJ or adjunctive BNJ) administration compared with corresponding baseline measurements (all values *P* < .05). But the Agg_max_ 7 d after washing in CYP2C19*1/*1 and CYP2C192/*2 genotypes were significantly higher than those at baseline, except for Agg_max_ in CYP2C19*1/*2 genotype. Agg_max_ values after BNJ or adjunctive BNJ clopidogrel therapy in three different CYP2C19*2 genotypes were significantly lower than those 7 days after washing (Table 2, all values *P* < .01). On the other hand, the results of an analysis of variance found that IPAs of Agg_max_ with 5-*μ*mol/L ADP stimuli at 4 hours, 24 hours, 3 days, and 7 days after clopidogrel administration in CYP2C19*2/*2 genotype were all much lower than those in CYP2C19*1/*1 genotype (Figure 2, all values *P* < .01), and IPAs of Agg_max_ with 5-*μ*mol/L ADP stimuli in CYP2C19*1/*2 genotype were between CYP2C19*1/*1 and CYP2C19*2/*2 (Figure 2). IPAs of Agg_max_ with 5-*μ*mol/L ADP stimuli 5 days after BNJ administration or 7 days after BNJ-combined clopidogrel had no significant difference among three different CYP2C19*2 genotypes. 

**Figure 3:**
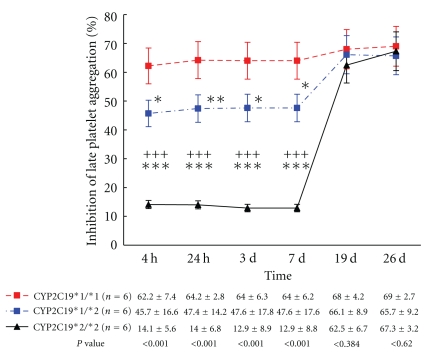
Inhibition of late platelet aggregation with 5-*μ*mol/L ADP between baseline and after therapy among the three *CYP2C19 *genotypes. The points represent the mean platelet inhibition; bars indicate standard deviations. *P* values and symbol as in Figure 2.

Significant reductions in Agg_late_ after any drug (clopidogrel or BNJ or adjunctive BNJ clopidogrel) administration were also observed in three different CYP2C19*2 genotypes, compared with their corresponding baseline measurements (all values *P* < .05). Likewise, Agg_late_ 7 days after washing in CYP2C19*1/*1 and CYP2C192/*2 genotypes were significantly higher than those at baseline, except for Agg_late_ in CYP2C19*1/*2 genotype (Table 2). Agg_late_ values in three different CYP2C19*2 genotypes were significantly different between 7 days after washing and 5 days after BNJ or 7 days after adjunctive BNJ clopidogrel therapy (Table 2, all values *P* < .01). IPAs of Agg_late_ with ADP stimulus are illustrated in Figure 3. IPAs of Agg_late_ were similar to IPA of Agg_max_ with 5-*μ*mol/L ADP stimulus after treatment among three different CYP2C19*2 genotypes.

**Figure 4:**
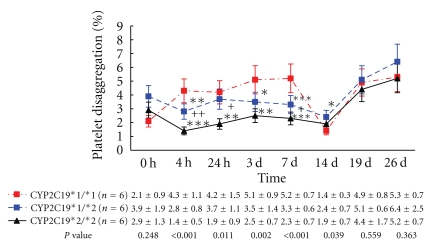
Platelet disaggregation with 5-*μ*mol/L ADP between baseline and after therapy among the three *CYP2C19 *genotypes. The points represent the mean platelet inhibition; bars indicate standard deviations. 0 h was at baseline. 14 d was 7 days after washing. The others as in Figure 2.

Baseline percentages of platelet disaggregation with 5-*μ*mol/L ADP stimuli had no significant difference among three different CYP2C19*2 genotypes. After clopidogrel therapy, percentages of platelet disaggregation were significantly different in three different CYP2C19*2 genotypes (Figure 4). The platelet disaggregation at 4 hours, 24 hours, 3 days, and 7 days after clopidogrel therapy was the lowest in CYP2C19*2/*2 genotype, compared with their corresponding clopidogrel therapy in CYP2C19*1/*1 and CYP2C19*1/*2 genotypes (all values; *P* < .05). The platelet disaggregation in CYP2C191/*2 genotype was between in CYP2C19*1/*1 and CYP2C19*2/*2 genotypes. 7 days after washing, percentages of platelet disaggregation in CYP2C191/*2 genotype was higher than those in CYP2C19*1/*1 genotype (*P* < .05). Percentages of platelet disaggregation 5 days after BNJ administration or 7 days after BNJ-combined clopidogrel had no significant difference among three different CYP2C19*2 genotypes (Figure 4).

### 3.3. Platelet Count, Platelet Activation, and Inflammation

**Figure 5:**
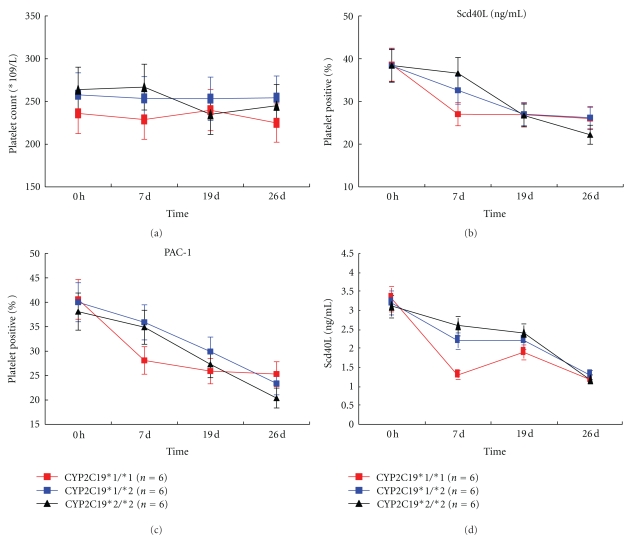
Effects of adjunctive naoxintong versus clopidogrel on platelet count (a), platelet activation (b, c), and inflammation (d) among the three *CYP2C19 *genotypes. The points represent the mean platelet inhibition; bars indicate standard deviations. *P* values and symbol as in Figure 2.

There were no significant differences in platelet count after treatment compared with corresponding baseline measurements, except for subjects in CYP2C19*2/*2 genotype (Table 3). Changes of platelet count were not significantly different among three CYP2C19*2 genotypes (Figure 5). CD62P, PAC1, and sCD40L were significantly reduced after clopidogrel administration or adjunctive BNJ-compared with corresponding baseline measurements (*P* < .01). After BNJ or BNJ combined clopidogrel, changes of these parameters were similar to changes of ADP-induced platelet aggregation in three different CYP2C19*2 genotypes (Figure 5).

## 4. Discussion

This ABNJC-CYP2C19*2 study is the first to demonstrate that adjunctive BNJ to clopidogrel can intensify platelet inhibition in volunteers with the CYP2C19*2 gene mutation including heterozygous (CYP2C19*1/*2) and homozygous for mutations (CYP2C19*2/*2). Furthermore, this study showed that adjunctive BNJ as compared with clopidogrel of 75 mg/day resulted in less platelet aggregation in CYP2C19*2 gene mutation. These results provide a rationale for further studies to assess whether adjunctive BNJ, as compared with other intensified regimens, provides long-term clinical benefits in patients with CYP2C19*2 gene mutation.

CYP2C19 plays an important role in the bioactivation of clopidogrel. The CYP2C19*2 allele is one of the mutations responsible for the phenotype known as poor clopidogrel metabolizer. The genetic polymorphism has been associated with a poor response to clopidogrel in healthy volunteers [21]. The clinical efficacy data mirror the effect of genetic polymorphisms on platelet function in both heterozygotes as well as homozygotes. Carriers of a CYP2C19*2 allele have been found to have an absolute reduction in platelet aggregation in response to clopidogrel that was 9 percentage points less than that of noncarriers [22]. In addition, medications metabolized by CYP450, such as omeprazole (an inhibitor of CYP2C19), have been shown to influence clopidogrel effect. Omeprazole has been associated with a lower efficacy of clopidogrel as assessed by the platelet reactivity index vasoactive-stimulated phosphoprotein [23]. 

TCM is an essential part of the health care system in several Asian countries and is considered a complementary or alternative medical system in most Western countries. Researchers are going to pay close attention to the regulation of Chinese materia medica on cytochrome P450 system [24]. Chan et al. [25] suggest that the interactions between Clopidogrel and *Angelica dahurica* or *Ginkgo biloba* or *Scutellaria baicalensis* can inhibit activities of CYP2C9 enzymes.

The ingredients of BNJ include Radix Astragali, Radix Angelicae Sinensis, Radix Paeoniae Rubra, Radix Salviae Miltiorrhizae, Rhizoma Chuanxiong, Semen Persicae, Flos Carthami, Resina Olibani, Myrrha, Caulis Spatholobi, Radix Achyranthis Bidentatae, Ramulus Cinnamomi, Ramulus Mori, Pheretima, Scorpio, and Hirudo. Wang's studies observed that Astragalus saponins are the essential component of BNJ confirmed by HPLC-MS [26]. The regulation of Astragalus saponins on cytochrome P450 system has not been reported. Xia's studies showed that Angelica sinensis Polysaccharides can modulate the activities of drug metabolism enzymes [27]. In our study, we observe that adjunctive BNJ can overcome an attenuation of the antiplatelet effect of clopidogrel in volunteers with the CYP2C19*2 gene mutation accompanied with qi deficiency and blood stasis constitution as demonstrated by all aggregometric and flow cytometric parameters obtained. Further researches should be undertaken to determine which ingredient or constituents of BNJ are with potential of CPY2C19 modulation.

### 4.1. Study Limitations

Although prospective, our study population is small, and they are not patients. Due to the difficulties in identifying the active constituents responsible for the modulation of CPY2C19 enzyme, prediction of BNJ-clopidogrel metabolic interactions is difficult. 

## 5. Conclusions

Among volunteers with the CYP2C19*2 gene mutation accompanied with qi deficiency and blood stasis constitution, adjunctive BNJ achieves intensified platelet inhibition as compared with clopidogrel of 75 mg/day. It needs to be evaluated whether the therapy could be translated into improved clinical outcomes. 

## Figures and Tables

**Table 1:** Subject characteristics.

	Age (year)	BMI (Kg/m^2^)	BP (mmHg)	Hb (g/L)	Platelet count (×10^9^/L)	FSG (mmol/L)	CGCCr (ml/min)	AST (*μ*/L)	ALT (*μ*/L)	TC (mmol/L)	CYP2C19*2 genotypes
1	33	21.8	130/70	134.0	257.0	4.93	92.1	36.6	38.6	5.3	CYP2C19*1/*1
2	36	21.0	118/70	136.0	204.0	5.19	89.3	33.3	37.3	5.0	CYP2C19*1/*1
3	38	19.8	118/80	153.0	295.0	4.69	87.6	38.7	38.9	5.4	CYP2C19*1/*1
4	48	22.1	120/70	152.0	189.0	4.69	88.2	34.6	36.4	5.2	CYP2C19*1/*1
5	35	21.7	110/70	167.0	240.0	4.68	90.2	33.2	33.3	4.1	CYP2C19*1/*1
6	36	24.5	102/80	125.0	231.0	4.96	84.6	37.8	38.7	4.6	CYP2C19*1/*1
7	42	22.8	120/80	126.0	269.0	6.33	87.5	32.9	39.2	5.4	CYP2C19*1/*2
8	35	23.9	116/70	135.0	273.0	4.88	91.6	39.6	37.7	3.6	CYP2C19*1/*2
9	32	21.3	120/80	154.0	271.0	5.44	92.3	33.7	38.1	5.6	CYP2C19*1/*2
10	34	23.5	106/74	133.0	218.0	6.96	111.6	36.5	38.8	5.0	CYP2C19*1/*2
11	34	20.1	108/70	164.0	217.0	4.68	119.8	37.9	36.7	3.4	CYP2C19*1/*2
12	37	23.4	120/80	156.0	298.0	4.48	116.6	38.8	37.7	4.1	CYP2C19*1/*2
13	36	18.4	120/80	153.0	288.0	4.98	87.9	38.1	37.3	3.9	CYP2C19*2/*2
14	43	21.1	120/70	152.0	293.0	6.6	86.3	40.0	39.4	3.9	CYP2C19*2/*2
15	45	24.2	124/84	145.0	298	5.39	87.7	38.2	39.0	5.2	CYP2C19*2/*2
16	35	21.6	100/70	139.0	217	5.55	102.3	36.9	34.6	4.3	CYP2C19*2/*2
17	41	20.6	108/84	149.0	203	4.19	106.6	37.5	35.7	4.2	CYP2C19*2/*2
18	39	23.1	116/80	145.0	284	5.32	95.4	36.7	37.5	5.1	CYP2C19*2/*2

Note: CGCCr is creatinine clearance rate calculated by Cockcroft/Gault formula.

**Table 2:** Comparison of platelet aggregation with 5-*μ*mol/L ADP at baseline and after therapy.

	Baseline	After single clopidogrel administration								7d after washing		5d after single BNJ administration		7d after BNJ combined- clopidogrel	

time	0 h	4 h	*P* value	24 h	*P* value	3 d	*P* value	7 d	*P* value	14 d	*P* value	19 d	*P* value	26 d	*P* value
Agg_max_, %															

CYP2C19*1/*1 (*n* = 6)	60.6 ± 5.6	23.3 ± 4.3	<.001	22.1 ± 2.3	<.001	24.4 ± 4.2	<.001	22.3 ± 2.8	<.001	70.4 ± 4.8	.012	23.3 ± 2.7	<.001	22.6 ± 1.2	<.001
													<.001*		<.001*
CYP2C19*1/*2 (*n* = 6)	66.9 ± 13.7	34.3 ± 4.5	.005	33.6 ± 3.2	.004	34.7 ± 3.5	.004	33.0 ± 3.3	.005	69.7 ± 4.1	.616	24.1 ± 5.2	.001	24. 7 ± 5.0	.001
													.001*		.001*
CYP2C19*2/*2 (*n* = 6)	62.9 ± 4.4	53.0 ± 3.0	.001	53.3 ± 2.1	.004	55.1 ± 2.6	.016	54.1 ± 3.5	.015	67.3 ± 3.8	.014	25.8 ± 3.8	<.001	22.7 ± 2.2	<.001
													<.001*		<.001*

Agg_late_, %															

CYP2C19*1/*1 (*n* = 6)	59.3 ± 5.8	22.2 ± 3.9	<.001	21.2 ± 2.4	<.001	23.1 ± 4.2	<.001	21.2 ± 2.3	<.001	69.5 ± 4.8	.012	22.1 ± 2.5	<.001	21.4 ± 1.2	<.001
													<.001*		<.001*
CYP2C19*1/*2 (*n* = 6)	64.3 ± 13.4	32.4 ± 3.2	.006	33.5 ± 3.5	.004	33.5 ± 3.6	.005	31.9 ± 5.3	.006	68.1 ± 4.4	.502	22.9 ± 5.1	.002	23.2 ± 5.2	.002
													.000*		.000*
CYP2C19*2/*2 (*n* = 6)	58.2 ± 6.6	55.0 ± 5.2	.003	52.25 ± 2.24	.007	53.7 ± 2.2	.016	52.9 ± 3.5	.021	66.0 ± 4.1	.021	24.6 ± 3.4	<.001	21.5 ± 2.1	<.001
													<.001*		<.001*

Note: values are presented as mean ± SD. The *P* values are for comparison to baseline. *The *P* values are for comparison to washing. ADP: adenosine diphosphate; Agg_max _: maximal platelet aggregation; Agg_late_: late platelet aggregation at 5 min.

**Table 3:** Comparison of platelet count, platelet activation, and inflammation at baseline and after therapy.

	Baseline	7 d after single clopidogrel administration	*P* value	5 d after single BNJ- administration	*P* value	7 d after BNJ- combined clopidogrel	*P* value
Platelet count (× 10^9^/L)							
CYP2C19*1/*1 (*n* = 6)	236.0 ± 37.9	228.7 ± 42.60	.172	239.7 ± 35.5	.315	224.7 ± 30.0	.093
CYP2C19*1/*2 (*n* = 6)	257.7 ± 32.85	253.5 ± 33.8	.627	251.3 ± 21.2	.474	254.2 ± 30.0	.489
CYP2C19*2/*2 (*n* = 6)	263.8 ± 42.2	266.7 ± 26.6	.890	234.8 ± 52.5	.037	245.0 ± 29.6	.085

CD62P platelet positive (%)							
CYP2C19*1/*1 (*n* = 6)	38.6 ± 2.7	27.0 ± 2.6	.001	26.9 ± 2.1	.001	26.0 ± 2.8	.001
CYP2C19*1/*2 (*n* = 6)	38.8 ± 1.9	32.6 ± 1.7	<.001	27.0 ± 2.2	<.001	26.2 ± 2.6	<.001
CYP2C19*2/*2 (*n* = 6)	38.4 ± 1.9	36.6 ± 2.0	.001	26.7 ± 1.9	<.001	22.2 ± 1.1	<.001

PAC-1 platelet positive (%)							
CYP2C19*1/*1 (*n* = 6)	40.6 ± 4.9	28.1 ± 2.6	<.001	25.9 ± 4.6	<.001	25.3 ± 4.5	<.001
CYP2C19*1/*2 (*n* = 6)	40.0 ± 2.3	35.9 ± 1.9	<.001	29.9 ± 2.2	<.001	23.4 ± 0.7	<.001
CYP2C19*2/*2 (*n* = 6)	38.1 ± 3.2	34.9 ± 2.0	<.001	27.3 ± 3.4	<.001	20.4 ± 1.2	<.001

Inflammatory marker sCD40L (ng/mL)							
CYP2C19*1/*1 (*n* = 6)	3.3 ± 0.9	1.3 ± 0.3	.001	1.9 ± 0.7	.001	1.2 ± 0.3	.001
CYP2C19*1/*2 (*n* = 6)	3.2 ± 0.7	2.2 ± 0.4	.002	2.2 ± 0.4	.002	1.3 ± 0.3	.001

CYP2C19*2/*2 (*n* = 6)	3.1 ± 0.2	2.6 ± 0.5	.007	2.4 ± 0.4	.007	1.2 ± 0.3	<.001

Note: values are presented as mean ± SD. The *P* values are for comparison to baseline. CD62p: platelet activation of the membrane marker P-selectin; PAC-1: antibody against activated glycoprotein IIb/IIIa; sCD40L: soluble CD40L.

**Table 4:** Characteristics of three different CYP2C19*2 genotyped subjects at baseline and the end of the test.

	CYP2C19*1/*1 (*n* = 6)		*P* value	CYP2C19*1/*2 (*n* = 6)		*P* value	CYP2C19*2/*2 (*n* = 6)		*P* value
	Baseline	The end		Baseline	The end		Baseline	The end	
Hb (g/L)	144.5 ± 15.5	145.7 ± 13.6	.428	144.7 ± 15.3	144.7 ± 16.3	1.000	147.2 ± 5.2	150 ± 6.9	.467
FSG (mmol/L)	4.9 ± 0.2	5.1 ± 0.6	.722	5.5 ± 1.0	5.2 ± 1.3	.437	5.3 ± 0.8	5.3 ± 0.7	1.000
ALT (*μ*/L)	37.2 ± 2.1	37.2 ± 2.3	1.000	38.0 ± 0.9	37.0 ± 3.3	.414	37.3 ± 1.9	37.2 ± 1.2	.921
AST (*μ*/L)	35.7 ± 2.3	36.3 ± 2.8	.171	36.6 ± 2.7	36.7 ± 1.8	.855	37.9 ± 1.2	36.8 ± 2.4	.309
CGCCr (ml/min)	88.67 ± 2.5	101.6 ± 9.9	.017	103.2 ± 14.3	92.0 ± 4.3	.099	94.4 ± 8.5	88.5 ± 5.5	.060
TC (mmol/L)	4.9 ± 0.5	4.8 ± 0.6	.782	4.5 ± 0.9	4.6 ± 0.7	.472	4.4 ± 0.6	4.2 ± 0.5	.398

Note: values are presented as mean ± SD. The *P* values are for comparison to baseline.
